# A Collaborative Initiative to Strengthen Sustainable Public Health Capacity for Polio Eradication and Routine Immunization Activities in the Eastern Mediterranean Region

**DOI:** 10.2196/14664

**Published:** 2019-10-29

**Authors:** Magid Al Gunaid, Faris Lami, Najwa Jarour

**Affiliations:** 1 Polio and Immunization Team Global Health Development/Eastern Mediterranean Public Health Network Amman Jordan; 2 Department of Community and Family Medicine College of Medicine University of Baghdad Baghdad Iraq

**Keywords:** GHD/EMPHNET, Global Polio Eradication Initiative, acute flaccid paralysis, vaccine-preventable disease, VPD, surveillance, Eastern Mediterranean region, EMR, Global Vaccine Action Plan, demand creation, microplans, polio transition

## Abstract

The many challenges in the Eastern Mediterranean region put the involved countries at risk of polio transmission and affect their ability to meet progress targets in eliminating vaccine-preventable diseases. The Global Health Development (GHD) and Eastern Mediterranean Public Health Network (EMPHNET) are working together on the project “Strengthening sustainable public health capacity in the Eastern Mediterranean region for polio eradication and routine immunization activities” with an overall goal of improving routine immunization, eradicating poliovirus, and controlling/eliminating or eradicating other vaccine-preventable diseases in the Eastern Mediterranean region. The aim of this manuscript is to describe the project and the achievements of GHD/EMPHNET over the last 3 years (2016-2018) to build effective surveillance and immunization systems in the Eastern Mediterranean region through the development of a sustainable and competent public health system to eradicate polio and control/eliminate vaccine-preventable diseases. This project assists the targeted Eastern Mediterranean region countries to build effective surveillance and immunization systems in an effort to expand their capacities to eradicate polio and control/eliminate other vaccine-preventable diseases. The project is streamlined with the Global Polio Eradication Initiative, the Centers for Disease Control and Prevention’s Strategic Framework for Global Immunization 2016-2020, and the Polio Eradication and Endgame Strategic Plan 2013-2018. The project also supports the Global Health Security Agenda by focusing on efforts to accelerate progress toward a world safe and secure from infectious disease threats. Project activities were designed to respond to countries’ needs and assist them in building their institutional and workforce capacity to effectively plan, implement, and evaluate activities to eradicate polio and strengthen routine immunization activities. The project activities covered a set of areas including surveillance of acute flaccid paralysis and other vaccine-preventable diseases, family and community engagement, workforce capacity building, improvement of data quality, management and use of information systems, use of polio assets to control/eliminate other vaccine-preventable diseases, support of countries to develop national strategies, piloting of innovative initiatives, program evaluation and accountability, and immunization strengthening. The project adopts the Global Polio Eradication Initiative strategies for assisting countries to strengthen routine immunization services, maintain highly sensitive acute flaccid paralysis surveillance, and sustain polio eradication functions.

## Introduction

The global efforts to eradicate polio since the launch of the Global Polio Eradication Initiative in 1988 have resulted in a 99% decrease in the number of reported wild polio cases [1]. The Eastern Mediterranean region aimed at achieving at least 90% routine immunization coverage nationally and 80% in every district by 2020 to be in line with the Global Vaccine Action Plan [2]. The Eastern Mediterranean region countries had a remarkable achievement in improving the routine vaccination coverage. However, several Eastern Mediterranean region countries experience immense challenges due to political unrest, complex emergencies and humanitarian crises, and socioeconomic hardships affecting this region. Thus, many of these countries witness a significant population movement, mainly because of forced displacement, flow of large numbers of asylum seekers, and refugees and migration of workers. These challenges have been associated with the collapse of health care systems; limited institutional and human resources; and disruption of surveillance, outbreak response, immunization systems, and other disease control measures [3]. The infrastructure damage, loss of trained personnel, and equipment shortages in the affected countries resulted in an increased vulnerability of the populations to communicable diseases [4,5].

The political crisis and wars in the region caused coverage of the Diphtheria, Tetanus, and Pertussis (DTP3) vaccine and third dose of oral polio vaccine (OPV3) to drop from 86% in 2010 to 80% in 2015, with wide inter- and intracountry differences. The quality of storage and cold-chain management, suboptimal compliance with recommended microplanning activities [6,7], insufficient funding, lack of transportation, and insecurity were the main challenges to the routine immunization programs in the region [8].

The challenges in the region put the countries at risk of polio transmission and affect their ability to meet progress targets in eliminating vaccine-preventable diseases (VPDs). Therefore, maintaining the minimal standardized performance of the Expanded Program on Immunization (EPI) in each of the targeted countries is essential to overcome the current and potential challenges. In this respect, strengthening of health systems requires improving accessibility and affordability of services as part of the universal health coverage efforts.

Countries in the Eastern Mediterranean region are at different levels of risk of polio transmission. Two countries within this region remain polio endemic (Afghanistan and Pakistan); other Eastern Mediterranean region countries are either ranked as very-high-risk (Syria, Yemen, and Somalia) or high-risk (Iraq, Sudan, and Libya) countries, and the remaining are classified as low-risk countries.

The Global Health Development (GHD) and the Eastern Mediterranean Public Health Network (EMPHNET) are working together on the project “Strengthening sustainable public health capacity in the Eastern Mediterranean region for polio eradication and routine immunization activities” with an overall goal of improving routine immunization, eradicating poliovirus, and controlling/eliminating or eradicating other VPDs in the Eastern Mediterranean region. The project is a 5-year funded project by the Centers for Disease Control and Prevention (CDC).

The aim of this paper is to describe the project and the achievements of GHD/EMPHNET over the last 3 years (2016-2018) in building effective surveillance and immunization systems in the Eastern Mediterranean region through the development of a sustainable and competent public health capacity to eradicate polio and control/eliminate VPDs.

## Project Overview

This project aims at assisting the Eastern Mediterranean region countries to build effective surveillance and immunization systems in an effort to expand their capacities to eradicate polio and control/eliminate other VPDs. Sustainability of services, on the other hand, is an essential part of the projects’ activities, where government involvement and community engagement play a major role in shaping national health policies to respond to the immunization-related challenges. Thus, the project is streamlined with the Global Polio Eradication Initiative, the CDC Strategic Framework for Global Immunization 2016-2020, the Polio Eradication and Endgame Strategic Plan 2013-2018, and the national policies and multiyear plans in relation to immunization. The project also supports the Global Health Security Agenda by focusing on efforts to accelerate progress toward a world safe and secure from infectious disease threats.

Project activities were designed to respond to countries’ needs and assist them in building their institutional and workforce capacity to effectively plan, implement, and evaluate activities to eradicate polio and strengthen routine immunization interventions. Accordingly, geographic targeting was initiated to prioritize the neediest populations in high-risk countries as well as countries that have specific weaknesses in their national systems but are not sufficiently addressed and do not receive adequate external support. The project activities covered a set of areas including surveillance of acute flaccid paralysis and other VPDs, family and community engagement, workforce capacity building, improvement in data quality, management and use of information systems, use of polio assets to control/eliminate other VPDs, supporting countries to develop national strategies, piloting of innovative initiatives, program evaluation and accountability, and immunization strengthening.

The project adopts the Global Polio Eradication Initiative strategies to assist countries to strengthen routine immunization services, maintain highly sensitive acute flaccid paralysis surveillance, and sustain polio eradication functions.

## Project Planning and Management

GHD/EMPHNET recruited and hired qualified staff at the headquarter and country levels to work with targeted countries for establishing connections with stakeholders in these countries. All core project staff hired were trained on EMPHNET policies and procedures, project strategy, objectives, and work plan.

Building on GHD/EMPHNET’s human resources assets in the region, Field Epidemiology Training Program graduates and residents were involved in the project’s activities to ensure sustainability and efficient implementation of the activities. Information sharing was guaranteed to all concerned health workers through the network of health care professionals, and data were timely provided to field workers.

Subregional and country-specific work plans to strengthen polio eradication and routine immunization were developed for six countries in the Eastern Mediterranean region. The subregional and country-specific work plans were developed by more than 20 experts and representatives from ministries of health of Jordan, Yemen, Iraq, Egypt, Afghanistan, and Somalia and EMPHNET key partners—World Health Organization (WHO) and United Nations Children’s Fund (UNICEF). The development of the subregional and country-specific work plans was preceded by providing countries with a set of reporting format, collecting a set of information-rich resources from the countries, analyzing the collected information and existing situation, and identifying the gaps and unmet needs in routine immunization services and polio eradication and the proposed solutions. In the years 2016 and 2017, three regional workshops were conducted and attended by a total 78 participants from different countries including Jordan, Yemen, Iraq, Egypt, Sudan, Lebanon, Morocco, Sudan, Tunisia, Afghanistan, Pakistan, and Somalia. The workshops aimed to strengthen polio eradication and routine immunization, build a pool of consultants, and analyze the training needs.

## Conducting Research to Inform Vaccine Preventable Disease Eradication, Surveillance, and Routine Immunization Activities

### A Cross-Sectional Serosurvey Among Children Aged <5 Years in Jordan

A serosurvey was conducted to assess population immunity in Jordan’s high-risk areas following the Middle East Polio outbreak response. GHD/EMPHNET, in collaboration with the CDC and Jordan’s Ministry of Health conducted a 5-day training for the data collectors, phlebotomists, and EPI managers to conduct the household serosurvey in 2016. The study sample included 479 children below 5 years of age from high-risk areas in the 12 governorates of Jordan and 277 children from Syrian refugee camps. The serosurvey provided data on immunization coverage in Jordan, determined the unimmunized pockets of children in high-risk areas, and estimated the population’s immunity levels to polio and other VPDs viruses. The seroprevalence among children in high-risk areas and refugee camps was 98% and 100%, respectively, for the three types of polio virus (types 1, 2, and 3). In addition, all children in Jordan, regardless of nationality, received three doses of OPV and three doses of inactivated polio vaccine during the first 9 months of life. The demand for these vaccines in Jordan was reported to be high. Results also indicated the Jordan Ministry of Health’s vigilance in locating and vaccinating high-risk populations has been successful in maintaining high levels of vaccination, which has, in turn, maintained high population immunity and averted polio outbreaks despite the influx of Syrian refugees. The study results primarily provided decision makers in Jordan with scientific evidence necessary to make informed decisions on effective interventions to prevent poliovirus outbreaks.

### Vaccination Coverage Survey in Iraq

One of the unmet needs in routine immunization identified by Iraq Ministry of Health is the unavailability of reliable data for immunization coverage in Baghdad Resafa and the recurrent outbreaks of VPDs over the past 3 years. Iraq experienced two cases of polio in 2014 and a measles outbreak in 2015-2016 and was more severely affected by the mumps outbreak in 2016. GHD/EMPHNET closely worked with Iraq Ministry of Health officials to a conduct immunization coverage evaluation survey in Baghdad Resafa. The survey protocol and tools were revised and finalized by GHD/EMPHNET and the WHO based on the standard WHO guidelines. GHD/EMPHNET supported a two-day training for 20 teams of data collectors. The survey provided data on the immunization coverage among children aged 12-23 months and identified the causes of never or delayed vaccination. The survey served as a baseline to monitor vaccination coverage and provide reliable and accurate data needed to strengthen the routine vaccination strategies and plans.

### Training Needs of Expanded Program on Immunization Staff

GHD, in collaboration with the CDC, conducted a training needs analysis to identify capacity building and the training needs of EPI staff in the five targeted countries: Morocco, Tunisia, Jordan, Egypt, and Sudan. The necessary tools were developed based on the Analysis, Design, Development, Implementation, and Evaluation Framework to help countries prioritize their training needs. GHD successfully conducted the training needs analysis workshop that was attended by 20 participants, 4 from each target country (Morocco, Tunisia, Jordan, Egypt, and Sudan). The participants included Directors, EPI Managers, EPI training and supervision officers, VPD surveillance officers, and EPI focal persons at the provincial/governorate level from each country. During the workshop, countries successfully presented their situation analysis and a summary of their country’s training needs.

### The Afghanistan Demand Creation Project

GHD/EMPHNET worked with the CDC on the activities of the Afghanistan Demand Creation Project (DCP). The DCP aimed to determine the effect of a three-pronged intervention on knowledge, attitudes, and practices toward routine immunization and polio, Supplementary Immunization Activities in Afghanistan, and determine the effect of the interventions on vaccine demand/uptake in Afghanistan. GHD/EMPHNET assisted in developing the training package for data collectors and supported the preintervention knowledge, attitudes, and practices survey and the development of interpersonal communication materials including materials for voice messages. In total, 1040 households from 20 districts (10 case districts and 10 control districts) were surveyed. The main findings of the baseline knowledge, attitudes, and practices survey were translated into local language and shared with field supervisors, which enabled them to address weaknesses in these areas during the community-based intervention. Following the completion of the preintervention baseline survey, GHD continued supporting and overseeing the community-based interventions in close collaboration with the Afghanistan field teams, Ministry of Public Health, and the CDC. GHD developed health communication training package material that was used to train Community Health Workers and community members on delivering effective and culturally appropriate immunization messages as well as developing voice messages. This was followed by successfully conducting the Communications Training of Trainers Workshop in 2017. A total of four DCP field supervisors in the eastern region and southern region and two national emergency operations center focal points were trained.

The DCP field supervisors conducted follow-up and field visits to the targeted districts. During these visits, supervisors collected feedback on the community intervention field activities, the mobile voice messages, and replacing the inactive phone numbers with the active phone numbers to ensure that voice messages reach the targeted audience. A total of 548 individuals were reached through the community meetings. The messages were broadcasted over 5 days per week over a month. The community-based intervention and broadcasting of mobile health messages were completed by the end of January 2018.

In total, 133 persons in Afghanistan were trained on data collection, analysis, and reporting (19 trained on advanced techniques in data management, analysis, and reporting; 98 trained on the EPI Management Information System database to improve the quality of EPI data; 6 trained on postintervention knowledge, attitudes, and practices survey; and 10 trained on data collection and using Epi Info 7 software).

## Strengthening Routine Immunization and Building the Capacity of Expanded Program on Immunization Staff

### Training Potential Consultants for Strengthening Routine Immunization

GHD/EMPHNET planned and successfully conducted a 6-day training workshop to build a pool of potential consultants for strengthening routine immunization at the regional level in 2016. The training workshop was attended by 37 participants selected from eight countries: six from Afghanistan, three from Egypt, five from Iraq, one from Jordan, one from Lebanon, five from Pakistan, eight from Sudan, and eight from Yemen. GHD/EMPHNET and representatives from the CDC, the WHO, UNICEF, the International Federation of Red Cross and Red Crescent Societies, and the Ministry of Health of Oman facilitated the workshop. A list of potential consultants was developed and added to the regional roster of potential consultants in the case of future deployment opportunities.

### Building the Capacity of National/Subnational Expanded Program on Immunization Staff on the Implementation of Routine Immunization Activities

GHD worked closely with the CDC on implementing training strategy to improve the competencies of EPI staff to contribute to the improvement of EPI activities and achieving national program objectives. The following activities were conducted to build the capacity of staff in targeted countries:

- GHD/EMPHNET supported the Yemen Ministry of Health to conduct an EPI situation analysis and identify implementation strategies for high risk areas. Three 5-days workshops for 54 supervisors of the Routine Immunization and Supplementary Immunization Activity services in 18 districts with low immunization coverage were conducted.
- GHD/EMPHNET supported training workshops to refresh knowledge and skills of vaccinators/health workers in Yemen to provide high-quality immunization services (eighteen 3-day refresher trainings workshops for 329 vaccinators/health workers in districts with low routine coverage).
- Cascade training of frontline health workers from 175 districts in 18 governorates of Egypt were trained on routine immunization.
- Training 25 master trainers in Jordan and 25 master trainers in Iraq on microplanning for routine immunization.
- Eleven training workshops on microplanning for routine immunization and development of microplans for 236 EPI staff from 115 health centers in 10 high-risk provinces and districts in Jordan.
- GHD in cooperation with the Ministry of Health successfully conducted 58 cascade training workshops and trained 1188 participants from 10 provinces in Iraq for routine immunization microplanning.

### Expanded Program on Immunization Communication Strategy and Implementation Plans

GHD supported Yemen and Iraq with the development of an EPI communication strategy and implementation plans. The identified strategic directions were as follows:

- Integrating Primary Health Care programs in health communication–related activities to improve EPI performance and meet the coverage challenges;
- Developing an integrated communication tools for all public health programs, which influence routine immunization/polio coverage;
- Communicating EPI health massages through education programs and curricula of schools and health institutes;
- Supporting studies and researches on communities’ attitudes and behaviors toward immunization;
- Evaluating and supervising all EPI activities in relation to communications and practices;
- Capacity building of health staff, with emphasis on EPI employees, in health communication;
- Unifying the health messages in relation to EPI at all levels to avoid discrepancies and misperceptions;
- Developing national information management tools in relation to health communication with emphasis on EPI;
- Announcing a national alliance for health communication with emphasis on EPI; and
- Ensuring the continuation of health communication at all levels of the health system during emergencies in relation to EPI and redesigning the messages based on local needs.

In Iraq, GHD maintained collaboration and communication with the Iraq Ministry of Health and UNICEF to do all necessary preparation to support the implementation of the activities. A workshop was conducted on dissemination and development of provincial level plans of the EPI communication strategy. A total of 45 participants from the Ministry of Health directors of immunization and health promotion in 16 provinces, Health Information Officers, and representatives from UNICEF attended the workshop. In January 2018, a workshop to develop the strategic direction and EPI communication strategy was held and attended by 28 persons.

### Supportive Supervision and Monitoring

GHD successfully trained a total of 43 managers/supervisors in Iraq and Yemen (23 Iraq Ministry of Health EPI managers/supervisors from 16 provinces, 20 EPI coordinators from 13 northern governorates, and central level EPI supervisors in Yemen) to form a team of supervisors/trainers to train district EPI focal point on supportive supervision, conduct in-service monitoring, and develop provincial and district Supervision & Monitoring (S&M) plans. In addition, GHD supported Yemen’s Ministry of Public Health and Population in conducting a national meeting on EPI monitoring and supervision that was attended by 50 participants from central and governorate levels. In the meeting, participants discussed main strengths, weakness, threats, and opportunities in relation to supervision and monitoring. The following issues were highlighted in the national meeting:

- The supervisory activities play an important role to augment the provision of immunization services.
- With exceptional geographic challenges and limited human resources, periodic intensiﬁcation of supervision is expected to pave the way for enhanced systematic vaccination outreach, which is now carried out six times per year.
- Intensiﬁcation of routine immunization and polio activities needs to focus more on remote and difficult-to-access populations that are traditionally underserved by routine services.
- Working with stakeholders for more engagement and community participation is essential to improve the vaccination coverage and maintain Yemen as a polio-free country.
- Coordination in implementing immunization campaigns is a vital element of planning and resource mobilization for periodic intensiﬁcation of routine immunization.
- Setting priorities, securing funding, and identifying additional partners for joint planning to achieve mutual beneﬁts are all vital elements that require well-designed supervision and monitoring system.

The meeting was concluded with an implementation plan for improving supervision at the national level to be followed with a national training for EPI coordinators from all governorates of Yemen with the support of GHD.

In a workshop held in Iraq, 15 persons attended the workshop and presented EPI supervision results, drafted an improvement plan, developed policy paper and updated S&M tools to assist the Ministry of Health in strengthening routine immunization S&M. On the other hand, GHD successfully conducted three refresher trainings for Afghanistan Ministry of Public Health provincial-level supervisors on conducting supportive supervision and monitoring to strengthen routine immunization performance. The 3-day trainings were conducted for a total of 45 EPI staff from 7 provinces. The strengthening of routine immunization performance activities has continued. Supportive supervision field visits were conducted for 15 provinces by country team in addition to provision of support for provincial supervisors to conduct S&M visits for 345 health facilities within 11 priority provinces.

### Appreciative Inquiry for Immunization Adaptation

GHD worked with the CDC to pilot an appreciative inquiry for immunization in Iraq to mobilize local communities and resources and build ownership among these communities with the aim of increasing vaccination coverage. The GHD team conducted a training workshop on appreciative inquiry for immunization adaptation for 30 health promotion EPI staff and other sectors in Baghdad to train and develop skills and abilities to facilitate national-, subnational-, and community-level appreciative inquiry workshops for full immunization and build local communities’ capacities to promote and engage households in full immunization activities and gain their commitment toward ensuring vaccination of every child, followed by two workshops to implement appreciative inquiry initiative. A total of 100 participants (40 in the Al Hur district and 60 in the Al Mahweel district) from the community, the Ministry of Health, nongovernmental organizations, and the development sector attended the workshops. The workshops aimed at developing aspirational goals, strategies, and breakthrough plans for full immunization and strengthen the leadership capacity to effectively mobilize communities toward full immunization in the two selected districts. The district workshops ensured enrollment of the whole system and stakeholders in full immunization initiative in the district, developed clear understanding and know-how about full immunization program, developed overarching vision by community and stakeholders in line with the national vision and strategy, and identified and committed key actions to be taken in communities. Furthermore, they defined the role, responsibility, and function of stakeholders in full immunization initiative and developed collaborative environment between demand and supply side to act together to achieve full immunization. GHD will continue working with the CDC and coordinate with the Iraq Ministry of Health on finalizing the Monitoring and Evaluation plan along with implementing the initiative and its evaluation.

## Strengthening Surveillance Systems

### Training on Acute Flaccid Paralysis and Vaccine-Preventable Disease Surveillance

GHD, in collaboration with the countries’ Ministries of Health, the CDC, and the WHO, supported the strengthening of the countries’ surveillance system for acute flaccid paralysis, measles, and other VPDs through capacity building activities. These activities aimed to enhance early detection and response to polio virus, measles, and other VPDs, thus supporting in sustaining polio eradication and reducing risks of measles and other VPDs outbreaks. A total of 71 persons (37 from Morocco and 34 from Iraq) were trained to be master trainers on acute flaccid paralysis and VPDs surveillance. A total of 1076 persons (311 from Iraq, 419 from Yemen, and 346 from Morocco) were trained on acute flaccid paralysis/VPD surveillance.

The training in Yemen aimed at introducing the community-based model in high-risk districts to improve the relationship between communities and the local health system through the technical and administrative link between the volunteers and health care providers and to enhance the reporting system and increase the sensitivity of the epidemiological surveillance, thus improving overall performance of acute flaccid paralysis surveillance. The training in Morocco aimed to address weaknesses, refresh knowledge, build capacity of the newly recruited officers at regional and provincial levels, train focal persons from major educational hospitals, and enhance the capacity of the Field Epidemiology Training Program graduates and residents to provide technical support, mentoring, coaching, and progress monitoring of acute flaccid paralysis surveillance activities against the regional acute flaccid paralysis operational plans and support improving key acute flaccid paralysis surveillance indicators, achieving and sustaining progress.

On the other hand, GHD developed a digital acute flaccid paralysis surveillance system including digital acute flaccid paralysis notification, investigation, and active surveillance forms in Morocco and installed it to the Ministry of Health server. The system was also further developed to generate reports and have a feature of sending email notifications directly to surveillance supervisors once cases are reported. GHD technical support will continue and the development of a mobile app of the system is planned once the system is ready and in use, and the mobile app design will be in synchronization with the system Web version.

### Assisting Egypt’s Ministry of Health in Establishing a Congenital Rubella Syndrome Surveillance System

GHD worked with Egypt’s Ministry of Health and Population (MOHP) to establish and support running congenital rubella syndrome surveillance system in Egypt. The congenital rubella syndrome surveillance system will provide the MOHP with surveillance data to monitor the impact of the national rubella vaccination strategy as well as the progress toward rubella/congenital rubella syndrome elimination and take necessary measures to reach targets of rubella elimination. The program was officially launched in Cairo on October 17, 2017, in an official ceremonial event attended by the Deputy Minister, the Director General of Communicable Diseases Control, the EPI Manager, and GHD/EMPHNET senior management alongside the central EPI team and the representatives of the targeted provinces.

### Supporting the Congenital Rubella Surveillance System in Afghanistan

As a joint activity with the CDC, GHD logistic support to the congenital rubella syndrome system was provided by contacting the congenital rubella syndrome project coordinator in Afghanistan who supported the implementation of study to determine the burden of congenital rubella syndrome in order to inform the initiation of routine immunization and prospective congenital rubella syndrome surveillance system in Afghanistan. GHD played a key role in facilitating communication, coordination, and progress follow-up of project activities with the Ministry of Health and the CDC through regular reporting. GHD provided a platform to further engage the Ministry of Health EPI staff in this activity. The congenital rubella syndrome coordinator was provided with capacity-building opportunity to support his work pertaining to cleaning datasets, rechecking/validating data for accuracy, and performing various analysis tasks on datasets. The project coordinator also attended the training workshop on Advanced Techniques in Data Management, Analysis, and Reporting conducted and facilitated by GHD in Afghanistan in June 2017.

## Increasing the Number of Programs Focusing on Outbreak Response, Systems Strengthening, and Workforce Development

### The Public Health Empowerment Program for Surveillance Polio Officers

As indicated by the Federal Ministry of Health of Sudan, Sudan has a shortage in trained human resources responsible for public health surveillance, and there is a need to empower the management and leadership skills for the workforce at the district levels. To bridge this gap, the Federal Ministry of Health planned to train health workers in 179 localities to build their capacity in areas of public health surveillance, leadership, management, and other public health skills to enable them to undertake additional tasks to support the health system. The Public Health Empowerment Program for Surveillance Polio Officers was accordingly developed to build the capacity of polio and EPI staff to equip themselves with competencies essential to public health surveillance and managerial skills. Thus, the program supported the implementation of polio transitional planning and assisted expansion of the technical capacity of polio program staff to fill gaps in manpower that are usually encountered at the district level for various public health services, without compromising their original roles and functions.

Moreover, GHD developed the curriculum for Yemen’s Public Health Empowerment Program to train governorate surveillance polio officers. GHD conducted an orientation workshop and a stakeholder’s workshop to build consensus on the Public Health Empowerment Program for Surveillance Polio Officers and enhance coordination among partners. The orientation meeting was attended by 39 officials including Directors of Health Offices, Local Council Representatives, and senior officials from the central level. The stakeholders’ workshop was attended by 24 officials and partners including the Minister of Health, Deputy Minister, General Director of Disease Control, other Ministry of Public Health and Population officials, and other partners such as the WHO and UNICEF. The workshop aimed at enhancing coordination among the development partners on the Public Health Empowerment Program for Surveillance Polio Officers in Yemen and to harmonize efforts with the Ministry of Public Health and Population, donors, and development partners for training at the governorate and district levels.

As a result of this program implementation, a total of 22 Public Health Empowerment Program for Surveillance Polio Officers mentors were trained in Yemen and 30 mentors were trained in Sudan. The program trained a total of 101 polio/EPI surveillance officers (83 in Sudan and 18 in Yemen), and it established a solid platform for capacity building at the subnational level, which is planned to target different key public health priorities.

### Vaccination Services at Refugee-Populated and High-Risk Areas in Jordan

GHD has maintained support to the vaccination center in the Zaatari camp for Syrian refugees. The center provides measles vaccines; OPV and inactivated polio vaccine; Bacillus Calmette–Guérin vaccine; and measles, mumps, and rubella vaccine to children below 2 years of age. The GHD support to the center maintained its engagement in the Syrian crisis health response and improved immunization coverage in the camp.

### Transition Polio Program Assets to Support Reaching Targets for Measles/Rubella Elimination in Egypt

GHD, in cooperation with the CDC, supported Egypt’s MOHP in strengthening measles and other VPDs surveillance and response through utilization of domestic polio assets for low population immunity for measles in three frontier governorates—Matrouh, Red Sea, and Aswan—and also through provision of evidence-based data and methods of reaching high-risk populations for surveillance and routine immunization. Orientation and training activities were conducted for the central three governorates and five district-level EPI staff as well as for community-level informants. GHD and the MOHP conducted a 1-day orientation for the central 10 EPI epidemiologists in their role to train, supervise, and monitor activities in selected governorates. In total, 149 EPI staff were trained from the three governorates on how to utilize skills and assets from polio eradication to accelerate measles elimination.

### Improving Routine Immunization Performance in Afghanistan

The continued technical support to the Ministry of Public Health throughout the project period included but was not limited to the collection and analysis of routine immunization coverage reports from provinces and the provision of feedback to the provincial and EPI teams in the eastern, northern, and southeastern regions. Furthermore, support was provided by conducting scientific analysis to inform action by different departments. Highlight of other support completed include training of the Monitoring and Evaluation department staff on the EPI Management Information System; maintenance and reinstallation of the database; participation in a comprehensive EPI performance review study; and data/information collection at national, provincial, and health facility levels. Also, GHD actively participated in the National Emergency Action Plan review meeting with the WHO, UNICEF, Gavi, the Vaccine Alliance, the CDC, and the Ministry of Public Health.

### Conclusions

The performed projects efficiently contributed to enhancing the acute flaccid paralysis and national surveillance systems in the targeted countries based on the improved indicators. The progress made in the efficient utilization of local resources and strong community engagement toward universal health coverage reveals the importance of building on successful programs to enhance the health system, and polio legacy is an example of this progress. Additionally, addressing high-risk areas with immunization services benefited the targeted communities and vulnerable groups in accessing the basic service package with improved health indicators in the areas of interventions.

In this regard, GHD maintained a successful collaboration with different stakeholders, mainly the CDC, and worked closely to plan, develop, and implement the activities of these projects. To accomplish the tasks associated with the activities, GHD and the CDC identified country-focal persons and introductions were made to further enhance collaboration and coordination. The team members within GHD and the CDC engaged in frequent conference calls and exchanged email communications to organize and coordinate meetings and joint country visits for technical support and training workshops in terms of the production of technical materials and activity plans. The CDC support visits to GHD also allowed face-to-face discussions on project activities, implementation, and long-term plans. Through this cooperation, the CDC project team was always receptive to each country’s perspectives in all discussions and provided support to ensure smooth implementation of activities. GHD also maintained and continued close coordination with ministries of health in Afghanistan, Egypt, Iraq, Jordan, Morocco, Somalia, Sudan, and Yemen. Furthermore, GHD involved key partners in joint meetings and continued coordination efforts to harmonize activities throughout the project implementation with UNICEF and the WHO.

## Abbreviations

CDC: Centers for Disease Control and Prevention
DCP: Demand Creation Project
DTP3: Diphtheria, Tetanus, and Pertussis
EMPHNET: Eastern Mediterranean Public Health Network
EPI: Expanded Program on Immunization
GHD: Global Health Development
MOHP: Ministry of Health and Population
OPV3: oral polio vaccine (third dose)
S&M: Supervision & Monitoring
UNICEF: United Nations Children’s Fund
VPD: vaccine-preventable disease
WHO: World Health Organization
